# MSD: Multi-stage deception for data privacy protection

**DOI:** 10.1371/journal.pone.0323944

**Published:** 2025-06-02

**Authors:** Tamer Abdel Latif Ali, Mostafa M. Elsherbini

**Affiliations:** 1 Computer Science Department, Arab Academy for Science, Technology and Maritime Transport, Aswan, Egypt; 2 Department of Software Engineering, Arab Academy for Science, Technology & Maritime Transport, Aswan, Egypt; 3 Electronics and Communication Department, Arab Academy for Science, Technology and Maritime Transport, Aswan, Egypt; Cardiff Metropolitan University - Llandaff Campus: Cardiff Metropolitan University, UNITED KINGDOM OF GREAT BRITAIN AND NORTHERN IRELAND

## Abstract

With the exponential growth of electronically transmitted and stored data, ensuring data privacy and security has become a fundamental challenge for organizations and enterprises. Traditional encryption methods have limitations, such as vulnerability to advanced attacks and high computational complexity, that lead to the exploration of complementary strategies like deception techniques for enhanced protection. These methods aim to mislead unauthorized users by presenting protected data as if it were authentic, but the attack resilience is still insufficient. Multi-stage deception (MSD) methods leverage multiple deception strategies, such as complement, swapping, and stack reversal, to improve data protection levels and resistance against decryption attempts. Combining these techniques addresses gaps in single-stage approaches and offers a more robust defense. The proposed MSD method incorporates a classification of encryption and deception techniques and introduces a novel evaluation approach targeting critical performance factors. A tailored pseudocode algorithm is designed to optimize deception for various attribute types, validated through simulations. Simulation results reveal that the MSD method achieves a 100% value change in the first stage and 92% in the second stage, with an overall accuracy exceeding 95%. These findings demonstrate the method’s effectiveness in elevating data protection levels while maintaining low computational complexity. The study highlights the potential of multi-stage deception as a powerful tool for safeguarding sensitive information, achieving superior performance in data security. By offering a scalable and adaptable framework, the MSD method addresses emerging challenges in data protection while setting the stage for further advancements.

## Introduction

Recently, the huge need to transfer and share huge data has prompted the use of different emerging technologies such as the sixth-generation (6G) mobile communication network, cloud computing, and blockchain technology leading to the relationship with database exchange [1]. So, the sensitivity and importance of information and data in the database system should be secured from corruption and unauthorized access and provide privileges that allow the objects included in the database to be clearly defined [2]. To address the data privacy concerns, end-users may choose to encrypt their data before storing or transmitting it [2]. Currently, database security is a highly discussed topic and an essential tool to ensure secure data storage and transmission. This security is primarily achieved through two key approaches: encryption and deception [3]. Encryption is a mathematical field focused on data transformation, converting data into an unreadable format using a secret key. There are three categories of encryption methods, [4]:

1. **Keyless methods:** These do not use any keys in the encryption process. Examples include hash functions and pseudorandom number generators.
2. **Single-key methods:** These use a single key for the transformation function. This key is typically assigned to an individual to protect data that only they can access.
3. **Two-key methods:** These use two related keys, known as public and private keys, for the transformation function.

Although encryption is highly effective against hacking attempts, hackers know that the data is encrypted and may use various techniques to decrypt it [5]. Furthermore, the limitations of previous methods, such as being time-consuming, compromising data integrity, increasing data size, offering low privacy levels, and being highly complex, motivate the use of deception. The deception method is beneficial because it keeps the hacker unsure about the authenticity of the data [5].

The deception approach is a cybersecurity approach that employs decoys to divert attackers from actual assets. For database security, this might include using fake databases, tables, or records to mislead attackers into believing they have accessed valuable information [6].

A well-known method is a negative database (ND), which stores information on what is prohibited instead of what is permitted. This approach is effective in preventing data theft or unauthorized access while allowing simplified and efficient data retrieval for authorized users [7]. However, the ND approach decreases the likelihood of a hacker confirming the authenticity of the data, though it significantly increases the size of the data due to the deception mechanism [7].

The SNDB technique deceives attackers by substituting sensitive attributes with their complements, supporting various attribute types while maintaining the original data size and enabling quick retrieval through a deception key. Conventional encryption methods face challenges, including vulnerability to sophisticated attacks and significant computational demands. In addition, some deception techniques seek to confuse unauthorized users by displaying protected data as genuine, yet their resistance to attacks remains inadequate and leads to enlarging the data size to make redundancy. In contrast, the proposed MSD method improves privacy robustness and deception levels by combining techniques like complementing, stack reversal, and swapping with low computational complexity through the same data size.

The rest of the article is organized as follows: The literature review section introduces a comprehensive review for the encryption and deception approaches. The methodology section outlines the proposed method and our work. The results and discussion section demonstrates the main findings of the proposed method and provides a detailed discussion of the highlighted results. Finally, the conclusion and future work are introduced.

## Literature review

The rapid expansion of digital technologies and the exponential growth of data have brought unprecedented challenges in ensuring data security and privacy. Organizations across industries are constantly threatened by sophisticated cyberattacks, prompting researchers to develop innovative techniques for safeguarding sensitive information. This literature review explores various encryption and privacy-preserving methods, highlighting their contributions, limitations, and potential for integration into comprehensive security frameworks. Additionally, it examines deception techniques as complementary strategies to enhance data protection by confusing and misleading attackers. By analyzing existing approaches and identifying critical gaps, this review lays the groundwork for proposing advanced solutions that address the evolving complexities of data security.

One notable technique is slicing, which employs vertical and horizontal partitioning on datasets to enhance correlations within attributes while breaking associations between columns [4]. This technique organizes properties into columns based on their correlations that enhances in balancing data utility and privacy [4]. Attribute-Based Encryption (ABE) has emerged as a powerful approach to ensure privacy in large-scale cloud storage systems. It enables data owners to define access policies that govern how users can decrypt specific datasets [5]. By encrypting data according to these policies, ABE ensures that only authorized users with matching attributes can access the information, making it an effective method to manage privacy in distributed environments. Identity-Based Encryption (IBE) further contributes to data security by using human identities, such as email addresses or IP addresses, as public keys. This simplifies the key management process while preserving the anonymity of both the sender and the recipient within a public key infrastructure. IBE eliminates the need for complex certificate management but relies on the secure mapping of identities to keys, which can become a vulnerability if compromised [5].

Homomorphic encryption represents a groundbreaking advancement that allows computations to be performed directly on encrypted data without requiring decryption [5]. In this way, the sensitive information remains secure throughout the computation process. However, its high computational cost often limits its practical application, particularly in large datasets or real-time processing scenarios [5]. Storage path encryption addresses data security by breaking large datasets into sequential segments and distributing them across multiple storage devices managed by different cloud service providers. This segmentation reduces the risk of data breaches by ensuring that no single provider has access to the complete dataset. While effective, this approach can introduce challenges related to data retrieval and synchronization [5].

Techniques like the Secured Map Reduce (SMR) model optimize data processing and security by employing randomized algorithms and perturbation techniques [8]. By enhancing the security of MapReduce processes, SMR ensures the protection of sensitive information during large-scale data analysis. However, its reliance on randomization may result in reduced efficiency in certain scenarios [9] and [10]. However, sometimes the term "encryption using hash functions" might refer to using hash functions in conjunction with encryption for purposes like digital signatures, password storage, or data integrity verification. The recipient can decrypt the hash with the sender’s public key and verify it against the hash of the received data to ensure authenticity and integrity [11].

Privacy-preserving methods such as the Blind Third Party (BTP) and Blind Peer Approach (BLP) provide innovative solutions to address privacy concerns in user interactions [12]. BTP minimizes the exposure of sensitive data by eliminating direct interactions between users and third parties, while BLP encrypts queries using the service provider’s public key, ensuring that even peers cannot access the data. The Integrated Blind Parties (IBPs) technique combines these methods to further enhance privacy, though the reliance on intermediary entities can introduce new vulnerabilities [12]. Enhanced BTP a novel element, a unique token, has been integrated into the existing BTP framework. Consequently, this proposed technique serves as a robust assurance against any breaches [13].

Enhanced methods such as the Improved Advanced Encryption Standard (AES) technique and specialized algorithms such as the Block Nested Loop (BNL) Skyline Algorithms also play a significant role in advancing data encryption [14] and [15], respectively. Improved AES introduces phased operations to optimize key generation and encryption processes, while BNL Skyline Algorithms assist in selecting the most appropriate encryption methods for specific security and privacy challenges. Lightweight cryptography techniques (LWCT) are designed for resource-constrained environments like the Internet of Things (IoT) [16]. These techniques provide efficient and secure data transmission, as demonstrated in applications such as oil spill detection. However, their simplified design can sometimes compromise robustness, making them less suitable for highly sensitive applications [16].

In addition to encryption techniques, deception techniques provided an improvement and strength in data protection without the need for encryption and retrieval process, thus contributing to the issue of time. has been significantly advanced by , which include creating traps, lures, and fake environments to detect, mislead, or analyze malicious activity. These methods are designed to protect valuable data and systems by confusing and trapping attackers. One innovative approach is the Negative Database Algorithm, which employs negative database conversion to generate numerous values instead of a single tuple [6]. These generated datasets populate the database, preventing malicious queries from accessing the actual data. This approach leverages the concept of creating false datasets that are indistinguishable from authentic data, thereby securing sensitive information through encryption and virtual database techniques [7]. Another notable technique is the Special Negative Database (SNDB), which further enhances privacy in big data environments. This method aims to deceive malicious users or hackers by substituting sensitive attributes with their complements [17]. As a result, attackers are unable to distinguish between the original data and the data transformed by the SNDB process, ensuring robust privacy protection. By introducing these deception techniques, researchers have provided additional layers of defense, complementing traditional encryption methods [17].

Despite these advancements, several gaps and limitations persist. While slicing and encryption techniques ensure data privacy, they often face challenges related to scalability and computational efficiency when applied to massive datasets. Homomorphic encryption, for instance, offers remarkable functionality but comes with a high computational cost that hinders its practical implementation. Identity-based encryption, while effective in preserving anonymity, may expose vulnerabilities if the underlying identity mapping is compromised. Similarly, lightweight cryptography methods, although optimized for IoT devices, often lack the robustness required for high-stakes environments. Furthermore, approaches like BTP and BLP depend heavily on trust assumptions, which may not hold in dynamic or adversarial settings.

This work aspires to contribute meaningfully to the evolving landscape of data security by harmonizing innovation with practicality. The proposed method offers several advantages: it does not increase the data size, applies security measures only to sensitive attributes, leaving the rest of the data unencrypted, and allows for simple and fast retrieval since the authorized user possesses the deception key and knows which attributes are secured. In contrast, the proposed MSD method employs various techniques, such as complementing, stack reversal, and swapping, to enhance privacy robustness and deception level with low computational complexity. To measure privacy robustness, a retrieving resistance factor (RRF) and an evaluation technique are proposed. The contributions in this article are:

1. Introducing a complementary classification for different encryption and deception techniques.
2. Enhancing the deception level by using multiple stages instead of single-stage, especially in the PL and RL.
3. Proposing a pseudocode algorithm of the MSD for each attribute type.
4. evaluating the privacy robustness of the MSD method by proposing RRF and an evaluation technique based on the most effective factors.
5. Results demonstrate the effectiveness and robustness of the MSD method with low computational complexity.

## Methodology

### Multi-stage deception (MSD)

**Fig 1:**
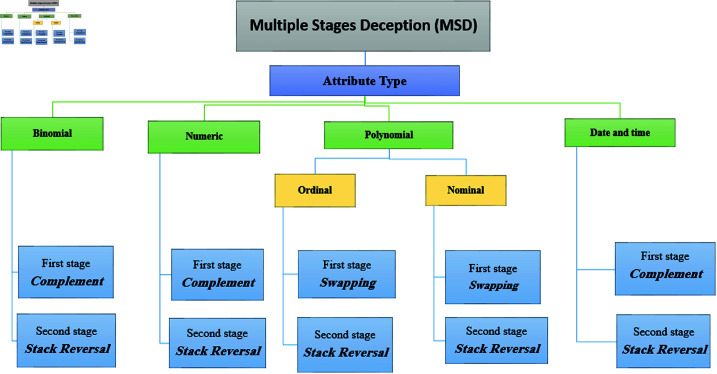
Attributes classification based on MSD.

As discussed in the previous section, the effectiveness of encryption and deception is highlighted by two key aspects: simplified and efficient data retrieval for authorized users and the preservation of the perception of reality. Moreover, deception can be implemented using a single-stage technique such as complementing, stack reversal, and swapping. In this paper, we propose combining multiple stages of deception techniques to enhance robustness while preserving the perception of reality. Fig. 1 illustrates the classification for all attribute types based on the MSD stage type, such as binomial, numeric, date, and polynomial. MSD is explained and evaluated for each attribute type; in addition, the retrieving resistance factor and the computational complexity estimation for each stage are addressed. Then, a pseudocode algorithm for each attribute type is introduced.

The robustness is defined as the resistance to retrieval, known as the RRF, which is represented by the probability of occurrence. Hence, the probability of occurrence is assumed to indicate the probability of retrieving the original data. In this way, the probability of data occurrence decreases; this means that the difficulty of retrieving data increases.

**Remark 1:** If the event A is a subset of sample space S, the probability of event A is:

$$P(A)=\frac{\text{ number of outcomes corresponding to event }(A)}{\text{ total number of outcomes in sample space }S}$$ (1)

In case of, two independent events *A* and *B*, the joint probability is

P(AB)=P(A)*P(B). (2)

Based on 2, the joint probability of the combination of two-stage deception techniques is the multiplication of the probability of data occurrence at each stage; hence, the value of the probability of data occurrence changes based on the deception technique. Additionally, the computational complexity for each stage of the deception technique is estimated by O(·).

1. **Binomial**
  All deception techniques could be applied with this attribute type. The complement and stack reversal were chosen as two deception stages. Hence, within this category, one subtype is binary, while the other is Boolean. Binary attributes entail two values: 0 and 1, whereas Boolean attributes consist of True or False, or two other values, the complement technique that represents opposites. The stack reversal means the reallocation of values in the stack. In stack reversal, the elements are rearranged so that the element at the bottom of the stack moves to the top, the second-to-bottom element becomes the second-from-the-top, and so on, until the original top element becomes the new bottom element.
**The RRF estimation:**
  Assume all possible changes are equally likely. So, the probabilities of complement and stack reversal are *P*_*C*_ = 0.5 and $P_{SR}=1/L_{att}$, respectively, where *L*_*att*_ is the number of records of the attribute. So, *RRF* is represented as:
  $$RRF=P_{C}*P_{SR}=1/(2*L_{att}).$$ (3)
**The computational complexity estimation**
  - 1st stage: *O*(1).
  - 2nd stage: *O*(*L*_*att*_).
  For this attribute, the deception method is carried out by putting the complement value of sensitive data, then stack reversal for the data records in this attribute. The above steps are summarized in the MSD Binomial algorithm below:
**Algorithm 1 MSD binomial.**

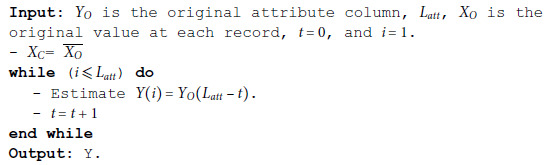

2. **Numeric**
  Numeric attributes can encompass integer or real numbers. So, all deception techniques are valid to apply. The complement and stack reversal are selected to be applied. In the first stage, the complement is carried out by partitioning each digit into *U*_*m*_ segments, and each value is replaced by the complement from the highest value *U*_*m*_. For example, the number of segments or values is 9, so the values 3 and 5 are replaced by 6 and 4, respectively. Then, the stack reversal as a second stage is applied to all records. As summarized in algorithm 2.
**The RRF estimation**
  As previously, all possible changes are assumed equally likely, in addition, any number assumed that it has a length of digits is *L*_*d*_. Also, each *L*_*d*_ is divided into non-sensitive digits *Z* and *S*_*d*_ sensitive digits. So, the probabilities of complement and stack reversal are $P_{C}=1/(L_{d}*U_{m})$ and $P_{SR}=1/L_{att}$, respectively. So, *RRF* is represented as:
  $$RRF=P_{C}*P_{SR}=1/(L_{d}*U_{m}*L_{att}).$$ (4)
**The computational complexity estimation**
  - 1st stage: $O(L_{d}-S_{d})$.
  - 2nd stage: *O*(*L*_*att*_).
**Algorithm 2 MSD numeric.**

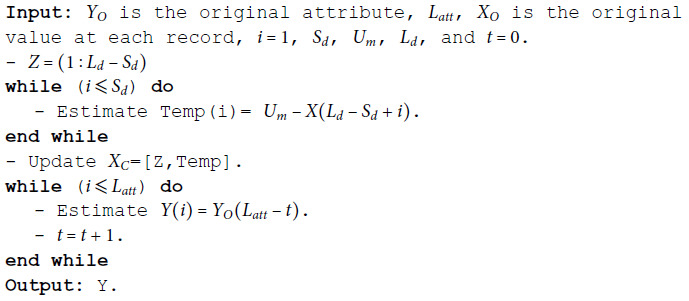

3. **Date and time**
  The property of the date is segmented into three components: year, month, and day. For day values, it converts the complement of each digit to *U*_*m*1_ equals 31. When representing a month, it complements the value to *U*_*m*2_ which equals 13. For year values, the first stage applied to the last digit of the year. The year values smaller than 2020 are complemented by adjusting the last digit to *U*_*m*3_ equals 9. While the year values greater than or equal to 2020 are complemented by adjusting the last digit to the current year, *U*_*m*4_. As summarized in algorithm 3. Regarding the time attribute, hours, minutes, and seconds are used for segmentation. For hour values, it is complemented to *U*_*m*5_ equals 24. When representing minutes or seconds, the values are complemented to *U*_*m*6_ equals 59. Then stack reversal is applied. As summarized in algorithm 4.
**The RRF estimation**
  As before, all possible changes are assumed to be equally likely. So, the probabilities of complement and stack reversal are $P_{C}=1/(U_{m1}*U_{m2}*U_{m3})$ and $P_{SR}=1/L_{att}$ , respectively. So, *RRF* for the date is represented as:
  $$RRF=P_{C}*P_{SR}=1/(U_{m1}*U_{m2}*U_{m3}*L_{att}).$$ (5)
  But also, the *RRF* for the time is represented as:
  $$RRF=P_{C}*P_{SR}=1/(U_{m5}*U_{m6}*U_{m6}*L_{att}).$$ (6)
**Algorithm 3 MSD date.**

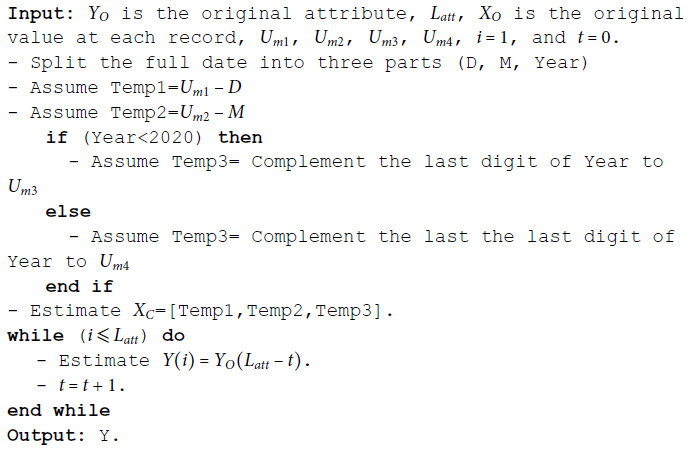

**Algorithm 4 MSD time.**

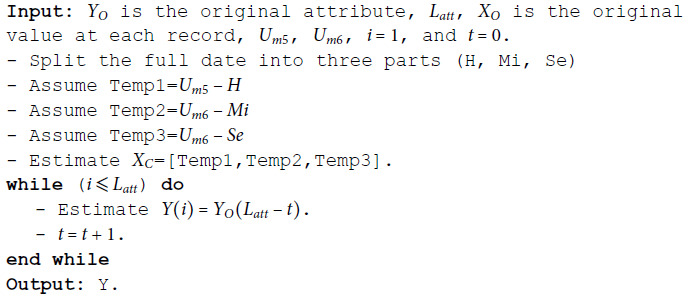

**The computational complexity estimation:**
  - 1st stage: *O*(3)–*O*(8).
  - 2nd stage: *O*(*L*_*att*_).
4. **Ordinal**
  When dealing with the switching approach, the number of categories is particularly essential in ordinal. Swapping is done to fool the bad user into believing that the data is genuine. Swapping is simple to do when there are an even number of categories. The first value will be swapped for the third, the second will be swapped for the fourth, etc. However, if there are an odd number of categories, we will first add a suitable value in the record at the end and then switch in an even manner. Then apply stack reversal as a second stage. As summarized in algorithm 5.
**Algorithm 5 MSD ordinal.**

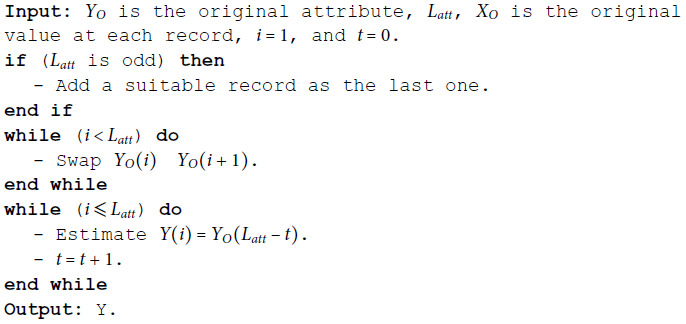

**The RRF estimation**
  As previously mentioned, all possible changes are assumed to be equally likely. So, the probabilities of swapping and stack reversal are $P_{S}=1/L_{att}$ and $P_{SR}=1/L_{att}$, respectively. So, the *RRF* is represented as:
  $$RRF=P_{S}*P_{SR}=1/(L_{att}^{2}).$$ (7)
**The computational complexity estimation**
  - 1st stage: *O*(*L*_*att*_/2).
  - 2nd stage: *O*(*L*_*att*_).
5. **Nominal**
  As in the ordinal case, except in the case of two correlated attributes such as name and gender, we need to apply deception techniques to two attributes. In the nominal situation, it is important to create a random list of values called *Y*_*r*_ which is used for swapping with original values. For example, a list of names will be formed if the values are the names of people. Then, at random, one value from this list will be replaced with the original, saving its index to the index list *Y*_*ind*_. The index list will help authorized users get the original data. Then stack reversal is applied as a second stage to enhance the deception impact. As summarized in algorithm 6.
**Algorithm 6 MSD nominal.**

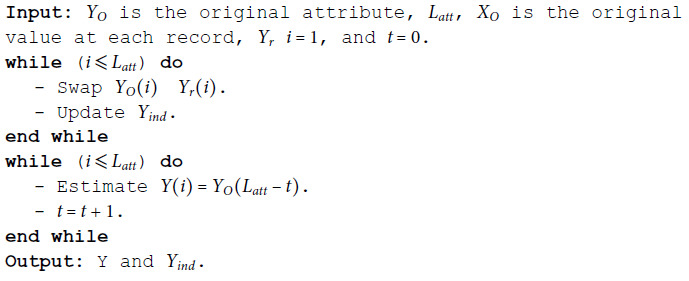

**The RRF estimation**
  As previously mentioned, all possible changes are assumed to be equally likely. So, the probabilities of swapping and stack reversal are $P_{S}=1/L_{att}$ and $P_{SR}=1/L_{att}$ , respectively. So, the *RRF* is represented as:
  $$RRF=P_{S}*P_{SR}=1/(L_{att}^{2}).$$ (8)
**The computational complexity estimation**
  - 1st stage: *O*(*L*_*att*_/2).
  - 2nd stage: *O*(*L*_*att*_).

### Proposed technique for evaluating privacy preserving algorithms

In this subsection, the author proposes a new evaluation technique for privacy preservation techniques based on eight factors taking into account three main categories. These categories are the data owner, authorized users, and unauthorized users. The eight factors will be introduced in the following subsections as qualitative measures.

1. **Data owner and authorized user evaluation factors**
  - *Protection level (PL) for data*
  - This factor measures the percentage of protection for the data of the data owner after applying the privacy-preserving technique against malicious attacks by ant and any privacy violation.
  - *Saving level of processing time (SLPT)*
  - This factor measures the percentage of processing time saving by applying the privacy-preserving technique to the data owner’s dataset. This savings level will be computed on the basis of the time consumed when applying the technique to the data set.
  - *Ease level (EL) of getting the original data*
  - This factor measures the percentage of how easy the privacy-preserving technique is to retrieve the original data. This factor is a very important factor for the data owner.
  - *Validity level (VL) of the technique for big data*
  - This factor measures the percentage of validity of the privacy-preserving technique that is to be applied to big data. This factor is very essential when dealing with large datasets.
  - *Simplification level (SL) of the technique*
  - This factor measures the percentage of how simple the privacy-preserving technique is for the data owner. The author here means the opposite of complexity. When the level of simplification is high, this means a low level of complexity which satisfies the data owner’s target.
  - *Integrity level (IL) of the data*
  - This factor measures the percentage of integrity for the data owner’s dataset after applying the privacy-preserving technique. The integrity means that the size of the dataset does not increase or decrease and the data does not violate any other challenge.
2. **Evaluation factors for unauthorized users**
  - *Unauthorized user’s deception level (UDL)*
  - This factor measures the percentage of how deceptive the privacy-preserving technique is to unauthorized users or hackers. The author here means that the hackers cannot differentiate between the original data and the data after applying the privacy-preserving technique.
  - *Resistance Level (RL) of decryption for unauthorized users*
  - This factor measures the percentage of the difficulty that the privacy-preserving technique is to be decrypted by unauthorized users or hackers. The author here means that hackers can or cannot get the original data according to the level of deception of the privacy-preserving technique.

## Results and discussion

**Fig 2:**
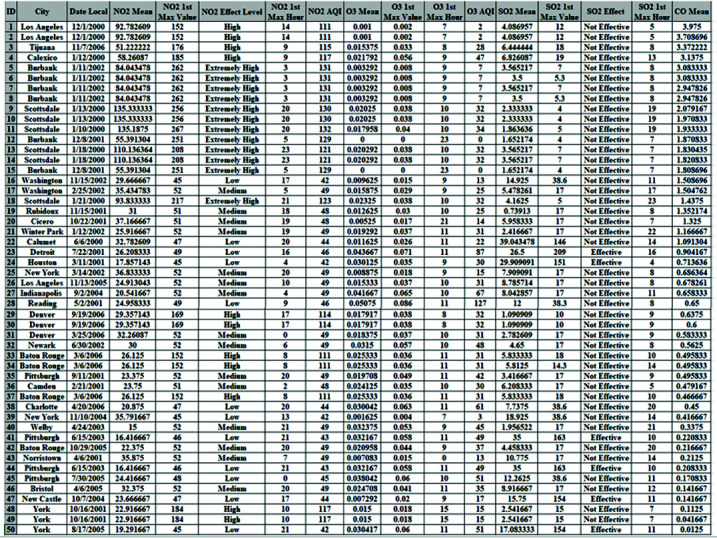
A sample of original dataset.

The dataset used in this article is related to pollution in the United States [18]. Organizing it in a format preferred by data scientists can be challenging. Consequently, data for four key pollutants–nitrogen dioxide (NO2), carbon monoxide (CO), ozone (O3), and sulfur dioxide (SO2)–were collected daily from 2000 to 2016 and compiled into a CSV file. The dataset includes twenty-eight fields in total, with each of the four pollutants (NO2, CO, O3, and SO2) having its own set of five columns. A sample of 50 records is taken from the whole dataset which consists of 1746661 observations as shown in Fig 2. The sensitive parameters assumed are the city, date local, NO2 mean, NO2 effect level, and SO2 effect.

**Fig 3:**
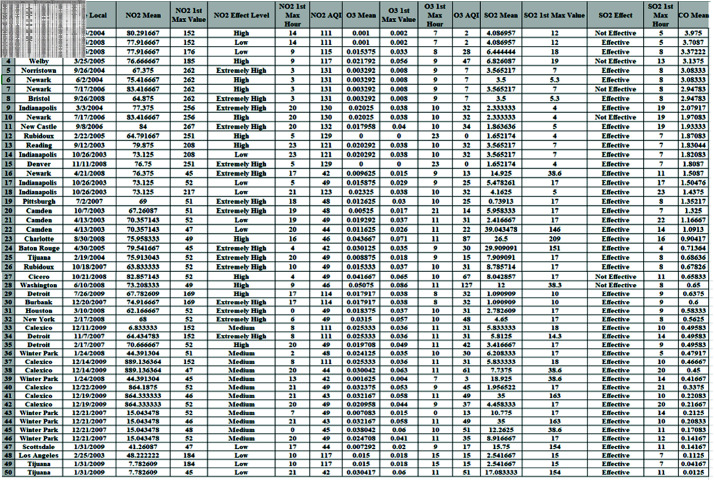
A sample of the dataset after applying MSD.

In this article, the results are assessed from two primary perspectives. The first perspective involves data visualization, which allows for a clear, graphical representation of the differences in the data throughout the different stages of the MSD method. This method helps to visually demonstrate how the data evolves from its original state through each stage of the transformation process. The second perspective focuses on quantitatively estimating the percentage difference between the original data and the data after each of the two stages of the MSD method as shown in Fig 3.

By estimating these percentage differences, we can precisely measure the extent of the changes induced by the MSD method, providing a concrete numerical evaluation of the transformation’s impact. Utilizing these two perspectives–visual representation and quantitative measurement–provides a comprehensive evaluation of the results, offering both an intuitive and precise understanding of how the MSD method affects the data. These two approaches ensure that the evaluation is thorough and multifaceted, capturing both the visual and numerical aspects of the data transformation.

**Fig 4:**
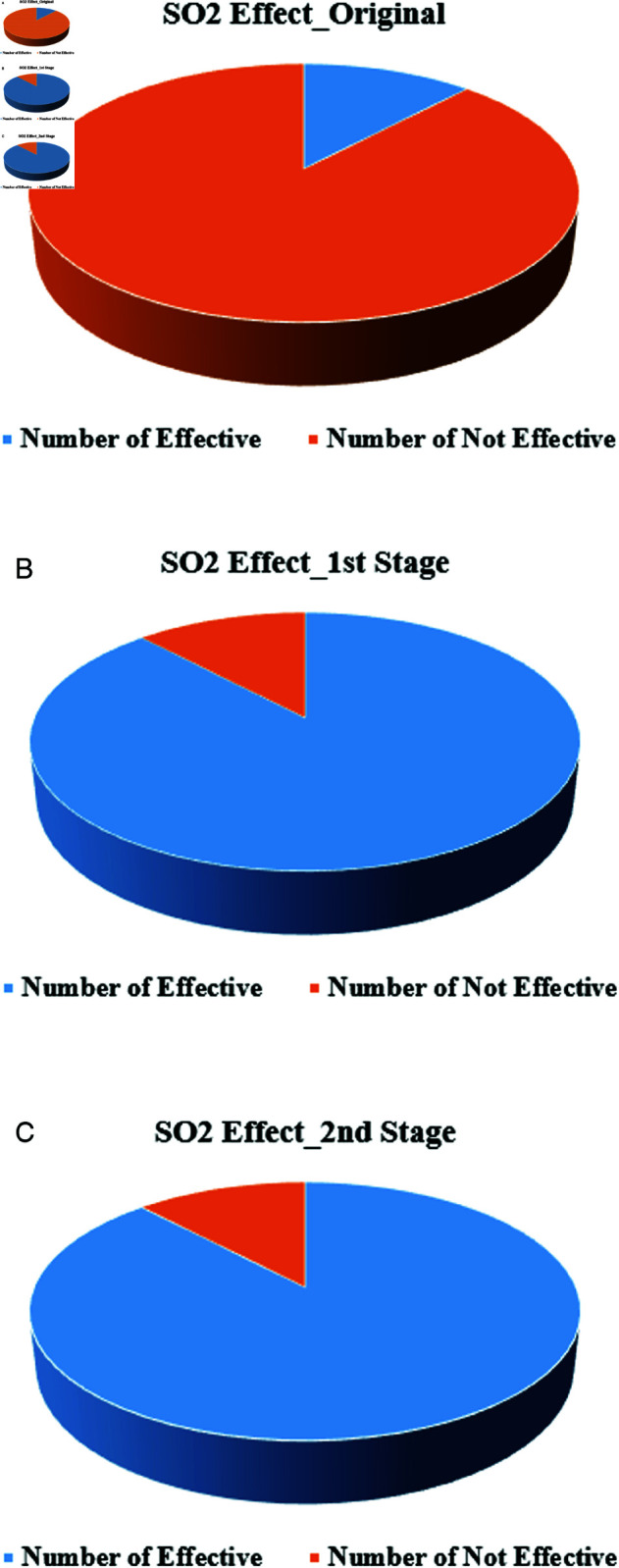
The change impact of SO2 Effect after MSD stages: (a) original data. (b) First stage impact. (c) Second stage impact.

**Fig 5:**
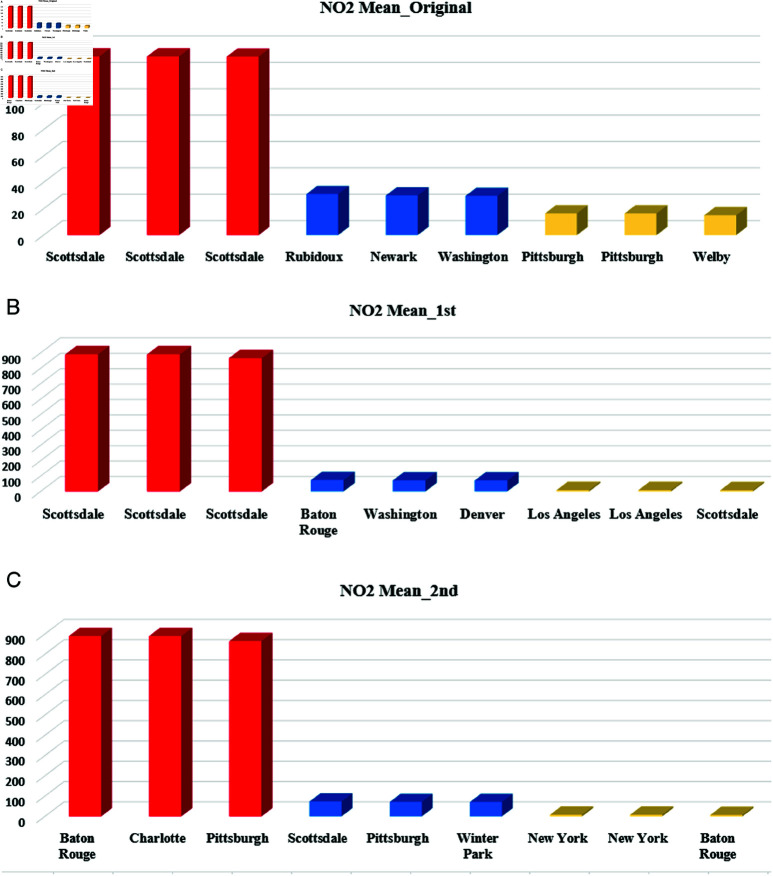
The distribution of NO2 Mean in the highest, middle, and lowest cities after MSD stages: (a) NO2 mean values in the original data. (b) NO2 mean values after the first stage. (c) NO2 mean values after the second stage.

**Fig 6:**
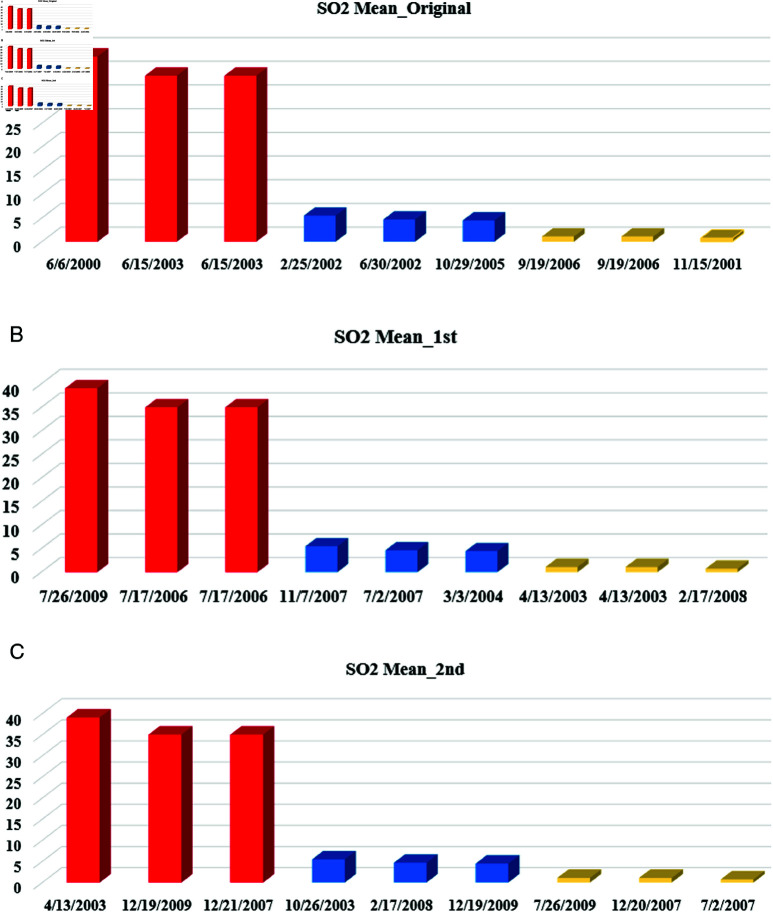
The distribution of SO2 Mean according to different dates after MSD stages: (a) SO2 mean values in the original data. (b) SO2 mean values after the first stage. (c) SO2 mean values after the second stage.

**Fig 7:**
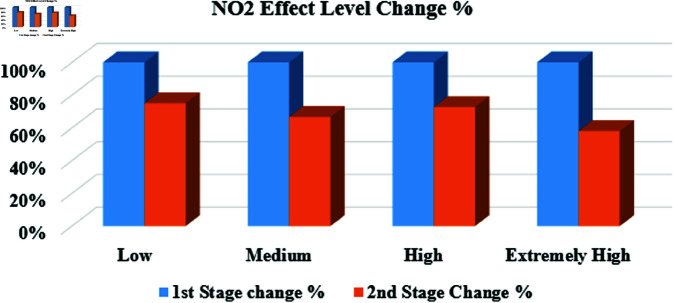
The percentage of change in NO2 effect Level between the MSD stages.

**Fig 8:**
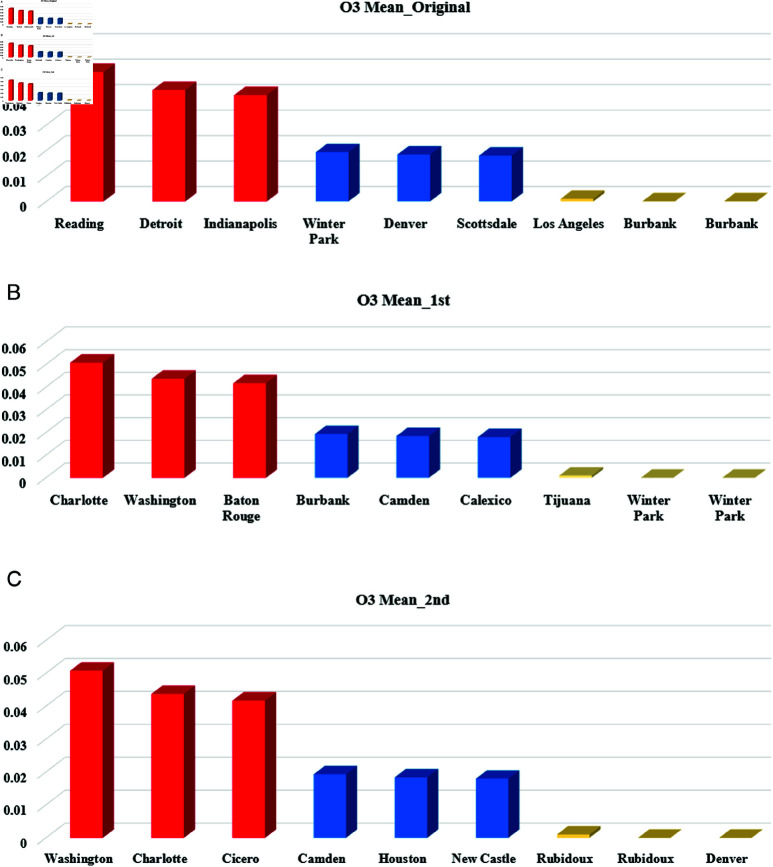
The distribution of O3 Mean in the highest, middle, and lowest cities after MSD stages: (a) O3 mean values in the original data. (b) O3 mean values after the first stage. (c) O3 mean values after the second stage.

1. **Binomial sensitive attribute**
  As detailed in the previous section, the binomial attribute consists of only two possible values, which means that the complement technique was able to completely alter the original data. In addition, the second stage was implemented to further enhance the level of deception. We applied the MSD method to the binomial-sensitive attribute (SO2 Effect) concerning cities with varying pollution levels. Fig 4 visually represents this progression, showing the changes in the pollution indicator as the data passed through the two stages of the MSD method. By the end of the second stage, the pollution indicators had been significantly altered, demonstrating the effectiveness of the MSD method in transforming the binomial-sensitive attribute and increasing the overall level of deception. In the first stage, the complement technique effectively reversed the values of the binomial attribute, resulting in a complete change from the original data as shown in Fig 4b. This transformation established the foundation for further modifications in the subsequent stage. The second stage, aimed at increasing the deception level, applied additional changes that compounded the initial alterations, resulting in a thoroughly modified dataset as shown in Fig 4c.
2. **Numeric sensitive attribute**
  After applying the MSD method to the numeric sensitive attribute (NO2 mean) using the complement technique in the first stage and stack reversal in the second stage, we observed a complete transformation of the original NO2 mean values across both stages. To illustrate the changes, we classified the values into three categories: highest, middle, and lowest, as depicted in Fig 5. Initially, Scottsdale had the top three NO2 mean values in the original dataset. However, after the second stage, the highest three NO2 mean values shifted to Baton Rouge, Charlotte, and Pittsburgh. This reclassification was also applied to the cities with the middle and lowest NO2 mean values, as shown in Fig 5.
3. **Date sensitive attribute**
  Fig 6 presents the measured SO2 mean values on various dates across different cities After applying the MSD method to the date-sensitive attribute (Date Local). Upon examining Fig 6, it becomes clear that the impact of deception is significant throughout the MSD stages when compared to the original data. As a result, the first stage (complement) is applied logically. For example, the range of dates over which the SO2 levels were measured spans ten years of the 21st century for the original, first, and second stages, as depicted in Fig 6b and 6c.
4. **Ordinal Sensitive Attribute**
  Fig 7 depicts the change in NO2 effect levels following the MSD stages. To assess this change, each effect level in the original data is compared to the new levels after applying the MSD stages on the ordinal sensitive attribute (NO2 Effect Level). After the first stage, all NO2 effect levels were completely altered. Subsequently, the second stage is implemented to further enhance the deception level, as demonstrated in Fig 7.
5. **Nominal Sensitive Attribute**
  After applying the two MSD stages to the sensitive attribute (City) in the original data, the city values are completely altered in both the first and second stages, as shown in Fig 8. Comparing the city values with the O3 mean across the original and the two stages reveals that the three city categories (High, Middle, and Low) have changed, as illustrated in Fig 8.

**Fig 9:**
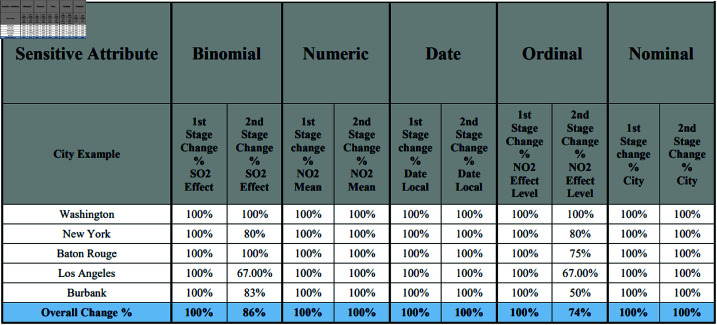
The overall change between the original data and the MSD stages among the five sensitive attribute types.

Fig 9 presents the percentage of the difference between the original data and MSD stages. For instance, in the binomial sensitive attribute, the pollution indicator in Washington and Baton Rouge was low based on the SO2 Effect in the original data. After applying the MSD method, the pollution indicator in these cities was changed to high. While in the original data, the NO2 mean values ranged from 15 to 135.33 parts per million. However, following the

MSD stages, the NO2 values varied from 6.83 to 889.13 parts per million, indicating a complete change in the analysis indicators. Ultimately, the overall percentage change in NO2 values was 100% for both the first and second stages, based on the sample average. Eventually, the overall percentage of change in the values of all five sensitive attributes is 100% in the first stage and 92% in the second stage.

**Fig 10:**
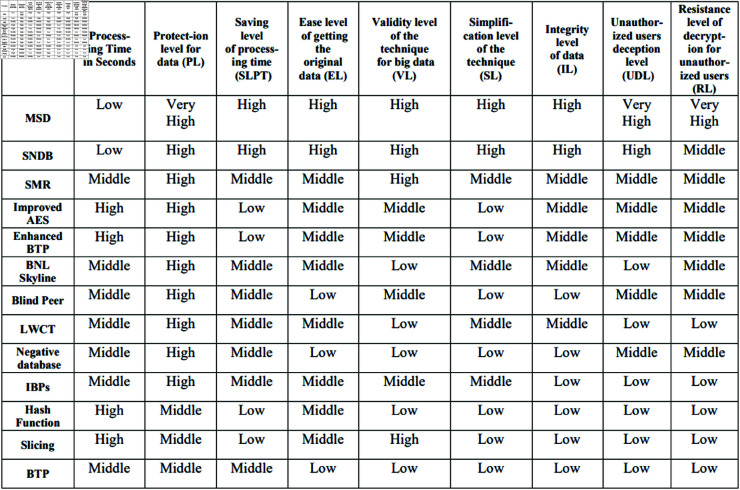
Privacy-preserving techniques evaluation.

Comparison among different privacy-preserving techniques according to the proposed evaluation technique, as shown in Fig 10. As illustrated, the grade of the evaluation ranges from low to very high. The MSD method got first place according to the overall grading of each evaluation factor. MSD method is superior to the SNDB technique in three evaluation

factors: PL, UDL, and RL. However, despite its advantages, the MSD presents some challenges:

- The complexity of the MSD method increases with additional stages of deception.
- Assessing the effectiveness of MSD for other types of attributes, such as images, audio, and video, remains a challenge.
- Applying the MSD to a nominal attribute may necessitate protecting a non-sensitive attribute due to the strong connection between sensitive and non-sensitive attributes.
- Addressing and validating the computational overhead associated with implementing multi-stage deception in real-world applications.

## Conclusion and future work

In this study, we presented a complementary classification for various encryption and deception techniques, providing a structured method for understanding and designing these security metrics. We proposed a novel evaluation that focuses on the most influential factors, ensuring a comprehensive evaluation of different methods. By enhancing the level of deception through a multi-stage approach, we significantly improved both the protection level of data (PL) and the resistance level against decryption by unauthorized users (RL). In addition, we developed a pseudocode algorithm for the Multi-Stage Deception (MSD) method designed for each attribute type, offering a practical guide for implementation. The results confirmed the effectiveness and robustness of the MSD method, demonstrating its superior capability in protecting sensitive information. These findings underscore the potential of multi-stage deception as a powerful tool in the privacy of data security. Despite the superiority of the proposed method, there are some limitations, such as applying MSD for other types of attributes, e.g., images, audio, and video. Also, validate the computational overhead associated with implementing multi-stage deception in real-world applications. Future research will further explore the applications of the MSD method in different real-world scenarios to validate its versatility and adaptability. Investigating the integration of machine learning algorithms to dynamically adjust the multi-stage deception process based on evolving threats will also be a priority.
